# Upregulation of lncRNA NONRATG019935.2 suppresses the p53-mediated apoptosis of renal tubular epithelial cells in septic acute kidney injury

**DOI:** 10.1038/s41419-021-03953-9

**Published:** 2021-11-01

**Authors:** Ying Ding, Dao-yang Zhou, Hong Yu, Tao Zhu, Feng Guo, Yang He, Xiu-liu Guo, Yong-jun Lin, Yu-jiao Liu, Yun-song Yu

**Affiliations:** 1 Department of Intensive Care Unit, Sir Run Run Shaw Hospital Xiasha Campus, Zhejiang University School of Medicine, 310018 Hangzhou, Zhejiang China; 2 Department of Emergency, Sir Run Run Shaw Hospital, Zhejiang University School of Medicine, 310016 Hangzhou, Zhejiang China; 3 Department of General Surgery, Sir Run Run Shaw Hospital, Zhejiang University School of Medicine, 310016 Hangzhou, Zhejiang China; 4 Department of Intensive Care Unit, Sir Run Run Shaw Hospital, School of Medicine, Zhejiang University, 310016 Hangzhou, Zhejiang China; 5 Department of Infectious Diseases, Sir Run Run Shaw Hospital, Zhejiang University School of Medicine, 310016 Hangzhou, Zhejiang China

**Keywords:** Cell biology, Molecular biology

## Abstract

Although increasing evidence has confirmed that the apoptosis of renal tubular epithelial cells (RTECs) is a crucial contributor to the onset and development of septic acute kidney injury (AKI), the pathological mechanism by which RTEC apoptosis is upregulated during septic AKI is not entirely clear. In this study, a rat model of septic AKI was induced by a cecal ligation puncture procedure or lipopolysaccharide (LPS) injection. Four differentially expressed long noncoding RNAs (DE-Lncs) in the rat model of septic AKI were determined using RNA-sequencing and verified by qRT-PCR. Among the four DE-Lncs, the expression level of lncRNA NONRATG019935.2 (9935) exhibited the most significant reduction in both septic AKI rats and LPS-treated NRK-52E cells (a rat RTEC line). The overexpression of 9935 suppressed cell apoptosis and p53 protein level in LPS-treated NRK-52E cells, and retarded septic AKI development in the rat model of septic AKI. Mechanistically, 9935 decreased the human antigen R (HuR)-mediated *Tp53* mRNA stability by limiting the combination of HuR and the 3′UTR region of *Tp53* mRNA in RTECs. The overexpression of HuR abrogated the inhibitory effect of pcDNA-9935 on the LPS-induced apoptosis of NRK-52E and rat primary RTECs. In conclusion, 9935 exerts its role in septic AKI by suppressing the p53-mediated apoptosis of RTECs, and this essential role of 9935 relies on its destructive effect on HuR-mediated *Tp53* mRNA stability.

## Introduction

Acute kidney injury (AKI) is one of the most common and life-threatening complications of septic patients. According to statistics, up to 60% of patients with sepsis have AKI, and patients with sepsis complicated by AKI have a prominently increased mortality compared with patients with sepsis alone [1, 2]. As the pathogenesis of septic AKI is incompletely understood, the current clinical therapy remains nonspecific. Therefore, clarifying the underlying pathogenesis of septic AKI is urgently needed for the development of effective treatment targeting sepsis-associated AKI.

The pathogenesis of septic AKI is traditionally attributed to decreased renal blood flow and acute tubular necrosis [3]. However, increasing evidence demonstrates that renal blood flow is normal or even increased in some specific patients and that few septic patients exhibit the features of acute tubular necrosis [4–6], suggesting that there must be other mechanisms driving the development of septic AKI. As early as 1999, Hotchkiss et al. [7] observed the upregulation of cell apoptosis in patients with sepsis. In recent years, with the continuous investigation of the pathogenesis of septic AKI, the crucial role of tubular cell apoptosis in septic AKI progression has been widely confirmed. Lerolle et al. [8] showed that tubular cell apoptosis was elevated in all septic AKI patients enrolled in their study, which was characterized by numerous activated caspase 3-positive cells in the kidney tubular. The treatment of caspase-3 inhibitor prevented apoptotic cell death and alleviated kidney failure in a murine model of septic AKI [9]. However, the underlying mechanism by which tubular cell apoptosis is upregulated in the septic kidney remains perplexing. One potential route may be through p53, a master regulator of cell apoptosis. As reported, the activation of p53 contributed to tubular cell apoptosis in cisplatin-induced AKI by targeting downstream pro-apoptotic genes [10]. The suppression of the p53 signaling pathway relieved lipopolysaccharide (LPS)-induced tubular cell apoptosis in a murine model of septic AKI [11]. Thus, suppressing p53-mediated tubular cell apoptosis may be a potential therapeutic target for septic AKI.

Long noncoding RNAs (lncRNAs), a kind of noncoding transcript longer than 200 nucleotides, are emerging as vital modulators in several biological and pathological processes [12]. The dysregulation of lncRNA expression has been observed in AKI, and several dysregulated lncRNAs have been proven to affect the progression of AKI. For instance, the plasma lncRNA TCONS_00016233 level was increased in septic AKI patients and showed the ability to be a diagnostic biomarker of septic AKI patients [13]. Moreover, lncRNA HOTAIR relieved the cecal ligation puncture (CLP)-engendered septic AKI in rats through the microRNA (miRNA)−34a/B-cell lymphoma-2 (Bcl-2) axis [14].

In the present study, by utilizing RNA sequencing, we determined a novel septic AKI-related lncRNA, NONRATG019935.2 (hereinafter referred to as 9935), which was significantly downregulated in the renal cortical of the rat model of septic AKI. Subsequently, we explored the function and underlying molecular mechanism of 9935 in the apoptosis of renal tubular epithelial cells (RTECs) in vitro and in vivo, expecting to provide new perspectives for the development of clinical therapeutic strategies against septic AKI.

## Materials and methods

### Septic AKI models

Sprague-Dawley rats (eight weeks old; male; Charles River, China) were used in our study. The animal ethics committee of Sir Run Run Shaw Hospital Xiasha Campus, Zhejiang University School of Medicine approved our experiments. The rat model of septic AKI was constructed by a CLP procedure or LPS injection. For the CLP procedure, the rats were anesthetized, and the cecum was exposed. The cecal ligation was performed using 3-0 sutures and then punctured with an 18-gauge needle. After that, a drop of feces was squeezed from the puncture hole [15]. The rats were sacrificed at 6 h (*n* = 6), 12 h (*n* = 8), or 24 h (*n* = 8) after CLP surgery. The rats that underwent sham surgery (the same procedure as the CLP surgery but without ligation and puncture) were set as the control (*n* = 8). For the LPS injection, LPS was intraperitoneally injected into the rats at a dose of 10 mg/kg, and the rats were sacrificed at 12 h (*n* = 5) or 24 h (*n* = 5) after LPS injection. The rats that received an intraperitoneal injection of the same volume of saline served as the control (*n* = 5). The kidneys of the rats were collected for further experiments.

To evaluate the influence of 9935 on septic AKI, lentivirus (LV)-9935 or its negative control (LV-NC) was generated by Hanheng Biotechnology Co., Ltd. (China) and then injected into the rats through the tail vein (injection dose: 1 × 10^9^/ml, 100 µl). Two weeks after injection, the LV-9935-treated rats were randomly divided into four groups: sham-LV-9935, CLP-LV-9935, control-LV-9935, and LPS-LV-9935. The LV-NC-treated rats were also randomly divided into four groups: sham-LV-NC, CLP-LV-NC, control-LV-NC, and LPS-LV-NC. Six rats were included in each group. All rats were sacrificed at 24 h after the sham/CLP procedure or LPS/saline injection, and the kidney tissues and blood samples of the rats were collected. The serum urea and creatinine (Cre) were detected using an urea assay kit and a creatinine assay kit (both purchased from Nanjing Jiancheng Bioengineering Institute, China), respectively.

### RNA sequencing by Illumina HiSeq

To identify differentially expressed lncRNAs (DE-Lncs) during septic AKI progression, the renal cortex of rats from the sham (*n* = 3), CLP 12 h (*n* = 3), and CLP 24 h (*n* = 3) groups was collected and sent to Beijing Biomarker Technologies Co., Ltd. (China) for RNA sequencing analysis.

### Renal tubular tissue isolation

The rats were sacrificed, and the right kidneys were collected. After removing the capsule and renal pedicle, the renal cortex was cut into small pieces and ground. Tissue homogenate underwent sequential filtration through 80‐mesh and 100-mesh screens and resuspended in saline. Then, the resuspension was centrifuged at 1500 rpm for 5 min, and the precipitate was the renal tubular tissues.

### Hematoxylin-eosin (H&E) staining

The kidney tissues of the rats were kept in paraformaldehyde for 48 h and then embedded in paraffin. Utilizing a rotary microtome, the tissues were cut into 4 µm-thick sections. Then, H&E staining was conducted using an assay kit (Boster Biological Technology Co., Ltd., China).

### Immunohistochemical staining

The protein levels of p53 and cleaved caspase 3 (c-caspase 3) in the kidney tissues of rats were determined using immunohistochemical staining. Renal tissue sections were orderly incubated with proteinase K, 3% H_2_O_2_, and 5% bovine serum albumin. The sections interacted with the primary antibody against p53 or c-caspase 3 for 12 h, followed by incubating with the secondary antibody for 1 h. The nuclei were stained with hematoxylin. The p53-positive or c-caspase 3-positive cells were visualized using a microscope.

### Cell culture and transfection

The rat RTEC line NRK-52E (Procell Life Science&Technology Co., Ltd., China) was cultured in DMEM supplemented with 5% fetal bovine serum (FBS). The rat primary RTECs were isolated according to the previous method [16] and contained in DMEM/F12 medium containing 10% FBS. All cells were incubated in an incubator with 5% CO_2_ at 37 °C. To induce the apoptosis of RTECs in vitro, NRK-52E cells/ rat primary RTECs were incubated with LPS (10 µg/ml) for 24 h.

Si-RNA targeting 9935 (si-9935), si-RNA targeting human antigen R (si-HuR), pcDNA 3.1 vector containing the cDNA sequences of 9935 (pcDNA-9935) or HuR, and their corresponding negative controls (si-NC and pcDNA-NC) were all synthesized by Shanghai GenePharma Co., Ltd (China). The cells were seeded in six-well plates at a density of 1 × 10^6^/well. For si-RNA transfection, 150 µl Opti-MEM medium containing 9 µl Lipofectamine® RNAiMAX reagent (Thermo Fisher, USA) was incubated with 150 µl Opti-MEM medium containing 30 pmol si-RNA for 5 min. The complex was added to the culture well and incubated with cells for 48 h. For pcDNA transfection, 125 µl Opti-MEM medium containing 7.5 µl Lipofectamine™ 3000 reagent (Thermo Fisher) was incubated with 250 µl Opti-MEM medium containing 5 µg plasmid and 10 µl P3000 reagent for 15 min. The complex was added to the culture well and incubated with cells for 48 h.

### Detection of cell apoptosis

TUNEL staining was performed on the kidney sections and NRK-52E cells to evaluate cell apoptosis. The entire process was carried out according to the manufacture’s instruction on the One Step TUNEL Apoptosis Assay Kit (Beyotime Biotechnology, China). Besides, the apoptosis of NRK-52E/rat primary RTECs was also measured using an Annexin V-FITC Apoptosis Detection Kit (Beijing Solarbio Technology Co., Ltd, China) and detected using flow cytometry.

### Quantitative real-time PCR (qRT-PCR)

Trizol reagent was purchased from Beyotime Biotechnology to extract the total RNA from the RTECs or kidney tissues. Reverse transcription was conducted with a PrimeScript™ RT Reagent Kit (Takara, Japan). qRT-PCR was performed using UltraSYBR Mixture (Cowin Biotechnology Co., Ltd, China). The relative expressions of the mRNAs and lncRNAs were calculated using 2^−ΔΔCT^ methods.

### Fluorescence in situ hybridization

To detect the subcellular localization of 9935, fluorescence in situ hybridization (FISH) was performed on NRK-52E cells and rat primary RTECs using a Cy3-labeled 9935 probe. The cells were seeded on the coverslips and orderly treated with 4% paraformaldehyde and 0.5% Triton X-100. Next, the coverslips interacted with the prehybridization solution (Guangzhou Ribo Biotechnology Co., Ltd, China), and then hybridized to the Cy3-labeled 9935 probe. DAPI was used to counterstain the cell nucleus. A confocal microscope was utilized to visualize the stained cells. Quantum dot FISH was performed on kidney sections to determine the subcellular localization of 9935 using a digoxin-labeled probe indirectly labeled with digoxin-antibody-conjugated quantum dots as previously described [17].

### RNA immunoprecipitation (RIP) assay

To determine the interplay between HuR and *Tp53/*9935, the RIP assay was conducted. The cell lysates of NRK-52E cells/rat primary RTECs were incubated with beads and anti-HuR/IgG antibody at 4 °C overnight. The enrichment of 9935 and *Tp53* mRNA in the immunocomplex was measured using qRT-PCR.

### Biotin pull-down assay

To determine the combination between HuR and 9935 in NRK-52E cells, the antisense oligomer affinity pull-down assay and RNA pull-down assay were performed. For antisense oligomer affinity pull-down assay, NRK-52E cells were transfected with antisense biotin-labeled DNA oligomers against 9935 (probe) or its negative control (NC, a sense biotin-labeled DNA oligomers against 9935) (all synthesized by Guangzhou Ribo Biotechnology Co., Ltd.), followed by the addition of streptavidin beads to extract the RNA-protein complex. The enrichment of HuR in the pull-downed complexes was examined using western blot. For RNA pull-down assay, cell lysates of NRK-52E cells were incubated with a biotin-labeled 9935 probe (sense) or its negative control (antisense, a biotin-labeled antisense RNA of 9935), followed by the addition of streptavidin beads to extract the RNA-protein complex. The enrichment of HuR in the pull-downed complexes was examined using western blot.

To determine the interaction between HuR and *Tp53* 3′ untranslated regions (UTR), the RNA pull-down assay was conducted using biotinylated-Tp53 3′UTR (Biotin-Tp53 3′UTR) as described above.

### Western blot

Protein samples of 25 µg extracted from the NRK-52E cells, renal cortex, or renal tubule were used for a single test. The experiments were conducted as previously described [18]. The primary antibodies used in this study were as follows: anti-c-caspase 3 (1:500; Abcam, UK), anti-caspase 3 (1:500; Abcam), anti-phospho-p53 (p-p53; 1:1000; Cell signaling Technology, USA), anti-p53 (5 µg/ml; Abcam), anti-HuR (1:1000; Abcam), anti-GAPDH (1:10000; Abcam), and anti-β-actin (1:5000; Abcam).

### Dual-luciferase reporter assay

The detection of *Tp53* promoter activity was conducted using the dual-luciferase reporter assay. Before detection, the sequence of the *Tp53* promoter was subcloned into the pGL3-basic plasmid. Well-grown NRK-52E cells were transfected with pcDNA-9935/si-9935/corresponding negative controls, followed by the recombinant vector and pRL-TK vector. Two days after transfection, the dual-luciferase reporter assay system (Promega, USA) was utilized to examine the luciferase activity. The relative luciferase activity of the recombinant vector was normalized by renilla luciferase activity.

To determine the specific region in which 9935 affected the *Tp53* transcript expression, the various regions of *Tp53* mRNA, namely full length, 5′UTR, 5′UTR + open reading frame (ORF), 3′UTR, and 3′UTR+ ORF, were subcloned into the pGL3 plasmid, respectively. The NRK-52E cells were transfected with si-9935/si-NC, recombinant vector, and pRL-TK vector, and the luciferase activity was measured.

### Detection of *Tp53* mRNA stability

To evaluate the influence of 9935 and HuR on *Tp53* mRNA stability, well-grown NRK-52E cells were transfected with pcDNA-9935/si-9935/si-HuR/pcDNA-HuR/corresponding negative controls, followed by actinomycin D (Act D, 5 µg/ml) treatment. The mRNA level of *Tp53* was measured by qRT-PCR at the indicated time points after Act D supplementation.

### Statistical analysis

We used GraphPad Prism 6.0 to conduct statistical analyses. Data were expressed as the mean ± standard deviation. Statistical significance between the two experimental groups was analyzed using Student’s *t*-test and *P* < 0.05 was set as the significance level. Pearson correlation analysis was employed to check the correlation between the HuR protein level and the *Tp53* mRNA level in the renal tubular tissues of the rat model of septic AKI. The correlation between DE-Lncs and pro-apoptosis genes was also examined by Pearson correlation analysis.

## Results

### Identification of DE-Lncs in CLP-engendered AKI

**Fig. 1:**
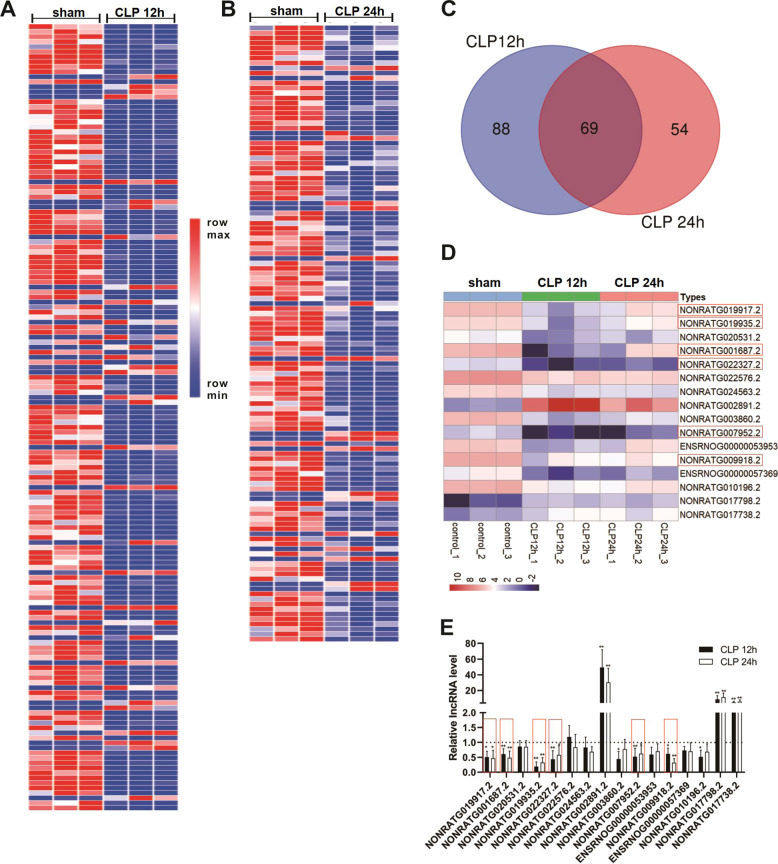
Identification of differentially expressed lncRNAs (DE-Lncs) in cecal ligation puncture (CLP)-engendered acute kidney injury (AKI). Clustered heat map of the DE-Lncs in renal cortical of **A** CLP 12 h (*n* = 3) and **B** CLP 24 h (*n* = 3). Rows represent lncRNAs and columns represent tissue types. The color scale runs from blue (low intensity) to white (medium intensity), to red (strong intensity). **C** There were 69 common DE-Lncs between CLP 12 and 24 h group. **D** The heat map of the 16 known lncRNAs among 69 common DE-Lncs. **E** qRT-PCR analysis of the expression levels of 16 known lncRNAs in the renal cortical of rats in each group (*n* = 5). *P < 0.05, **P < 0.01 vs. sham group.

A rat model of septic AKI was first constructed through CLP surgery. H&E staining confirmed that compared with the sham group, the CLP rats exhibited a distinct kidney impairment, which was characterized by tubular vacuolization, edema, and epithelial cell shedding (Fig. S1). Then, lncRNA sequencing found that relative to the sham group, 157 lncRNAs were differentially expressed in the renal cortical of rats from the CLP 12 h group (Fig. 1A) and 123 lncRNAs were differentially expressed in the renal cortical of rats from the CLP 24 h group (Fig. 1B). Among them, 69 lncRNAs were common DE-Lncs in the CLP 12 and 24 h groups (Fig. 1C), and 16 of them were sequences known. Subsequently, qRT-PCR was performed to examine the expression levels of the 16 known lncRNAs in the renal cortical of CLP rats. The results in Fig. 1D, E showed that 11 lncRNAs exhibited statistically changed expression levels and the variation trends were consistent with the sequencing results. Among these lncRNAs, six were distinctly downregulated in both the CLP 12 and 24 h groups. Owing to the low background levels of NONRATG007952.2 and NONRATG022327.2 expressions (Fig. 1D), we selected NONRATG019917.2 (hereinafter referred to as 9917), NONRATG001687.2 (hereinafter referred to as 1687), 9935, and NONRATG009918.2 (hereinafter referred to as 9918) for the follow-up studies.

### Effect of 9935 on LPS-induced RTEC apoptosis in vitro

**Fig. 2:**
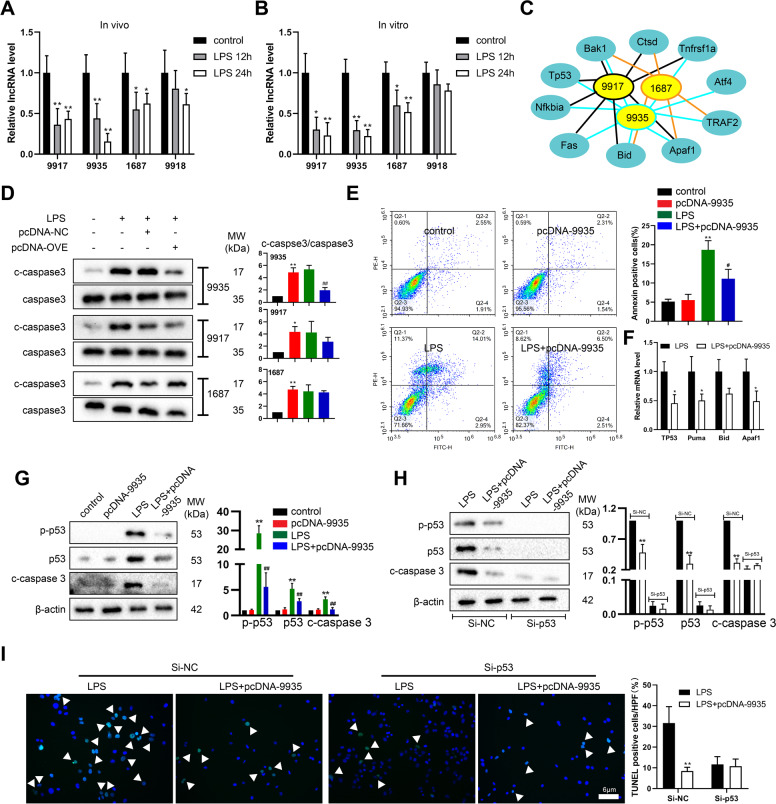
Effect of lncRNA NONRATG019935.2 (9935) on LPS-induced renal tubular epithelial cell (RTEC) apoptosis in vitro. The expression levels of NONRATG019917.2 (9917), NONRATG001687.2 (1687), 9935, and NONRATG009918.2 (9918) in the **A** renal cortical of control (*n* = 5), LPS 12 h (*n* = 5), and LPS 24 h (*n* = 5) rats; and **B** rat RTEC line NRK-52E treated with or without 10 µg/ml LPS (12 or 24 h). *P < 0.05, **P < 0.01 vs. control. **C** The correlations between 9917/9935/1687 and apoptosis-related genes were analyzed by Pearson correlation analysis. *Bak1* B-cell lymphoma 2-antagonist/killer 1, *Ctsd* cathepsin D, *Tnfrsf1a* tumor necrosis factor receptor superfamily member 1A, *Atf4* activating transcription factor 4, *Traf2* Tnf receptor-associated factor 2, *Apaf1* apoptotic peptidase activating factor 1, *Bid* BH3 interacting domain death agonist, *Fas* Fas cell surface death receptor, *Nfkbia* nuclear factor-kappa B inhibitor alpha, *Tp53* tumor protein p53. **D** The cleaved caspase 3 (c-caspase 3) protein level was measured in LPS-treated NRK-52E cells transfected with pcDNA-9935/9917/1687 overexpressing vector (OVE) or corresponding negative control (pcDNA-NC) using western blot. Caspase 3 was served as a control. *P < 0.05, **P < 0.01 vs. control; ##*P* < 0.01 vs. LPS + pcDNA-NC. **E**–**G** NRK-52E cells were transfected with/without pcDNA-9935 and then treated with or without LPS. **E** Cell apoptosis was measured by flow cytometry. **P < 0.01 vs. control; #*P* < 0.05 vs. LPS. **F** The mRNAs levels of *Tp53*, Bcl-2 binding component 3 (*Puma*), *Bid*, and *Apaf1* were measured by qRT-PCR. *P < 0.05 vs. LPS. **G** The protein levels of phospho-p53 (p-p53), p53, and c-caspase 3 were measured using western blot with β-actin as an internal control. **P < 0.01 vs. control, ##*P* < 0.01 vs. LPS. **H**, **I** NRK-52E cells were divided into four groups: si-NC + LPS, si-NC + LPS + pcDNA-9935, si-p53+LPS, si-p53+LPS + pcDNA-9935. **H** The protein levels of p-p53, p53, and c-caspase 3. **P < 0.01 vs. LPS + si-NC. **I** TUNEL staining was performed to determine cell apoptosis. Triangles: TUNEL-positive cells (green). The nuclei were counterstained with DAPI. **P < 0.01 vs. LPS + si-NC.

Subsequently, the expression levels of 9917, 9935, 1687, and 9918 were also determined in the LPS-treated rats and the LPS-treated rat RTEC line NRK-52E. As depicted in Fig. 2A, B, the expression levels of 9917, 9935, and 1687 were significantly decreased in response to the stimulation of LPS both in vivo and in vitro, hinting at their potential role during septic AKI progression. Given the vital role of RTEC apoptosis in the septic AKI progression, the correlation between the 9917/9935/1687 and apoptosis-related genes was analyzed, and the results showed that all of them were negatively correlated with the pro-apoptosis genes (Fig. 2C). Moreover, 9935, 9917, and 1687 were separately overexpressed in LPS-treated NRK-52E cells and 9935 overexpression most clearly downregulated the c-caspase 3 protein level (Fig. 2D) (the transfection efficiency of pcDNA-9935 was shown in Fig. S2A). In response to 9935 overexpression, the apoptotic cell numbers decreased, the mRNA levels of the downstream target gens of p53 were suppressed, and the protein levels of p-p53, p53, and c-caspase 3 were reduced in the LPS-stimulated NRK-52E cells (Fig. 2E–G). Considering the above data, we proposed a hypothesis that 9935 could influence the development of septic AKI by modulating the p53-mediated apoptosis of RTECs. Consistent with our speculation, the silencing of p53 reduced the c-caspase 3 protein level and TUNEL-positive cell numbers in LPS-stimulated NRK-52E cells, and further 9935 overexpression failed to affect the inhibitory effect of p53 knockdown on cell apoptosis (Fig. 2H, I).

### Effect of 9935 on sepsis-induced kidney injury in vivo

**Fig. 3:**
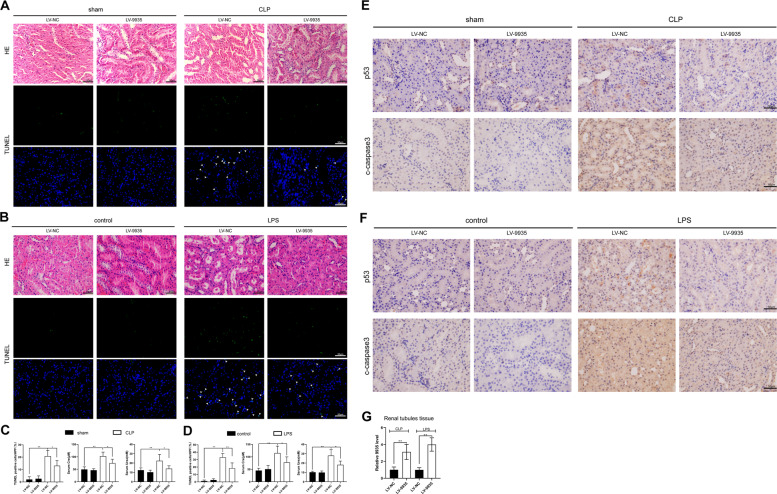
Effect of lncRNA 9935 on sepsis-induced kidney injury in vivo. The rat model of septic AKI was constructed by a CLP procedure or LPS injection. Two weeks before the modeling, lentivirus (LV)-9935 or its negative control (LV-NC) was injected into rats via the tail vein. *n* = 6 in each group. **A**, **B** Representative images of hematoxylin-eosin (H&E) staining and TUNEL staining performed on kidney sections of rats. **C**, **D** The quantitation of TUNEL staining was expressed as the mean number of TUNEL-positive cells in ten high-power fields (HPF). The serum levels of creatinine (Cre) and urea were measured by assay kits. **E**, **F** Representative images of immunohistochemical staining for p53 and c-caspase 3 performed on kidney sections of rats. **G** qRT-PCR analysis of the 9935 expression level in the renal tubules of rats. *P < 0.05, **P < 0.01.

The effect of 9935 on septic AKI was evaluated in vivo. 9935 was overexpressed in rats through the tail intravenous injection of LV-9935 and then the rat model of septic AKI was constructed by CLP procedure or LPS injection. First, the results of qRT-PCR proved that LV-9935 injection successfully enhanced 9935 expression in the renal tubules of rats (Fig. 3G). Second, we observed that 9935 overexpression greatly relieved kidney injury in septic AKI rats, which was characterized by lessened tubular vacuolization, reduced epithelial cell shedding, downregulated cell apoptosis (Fig. 3A–D), and decreased serum levels of Cre and Urea (Fig. 3C, D). Third, similar to the in vitro data, 9935 overexpression also suppressed the expression levels of p53 and c-caspase 3 in the kidneys of septic AKI rats (Fig. 3E, F). The aforementioned results hinted that 9935 overexpression downregulated the apoptosis of RTECs and alleviated sepsis-engendered kidney injury in vivo.

### 9935 overexpression downregulated the mRNA stability of *Tp53*

**Fig. 4:**
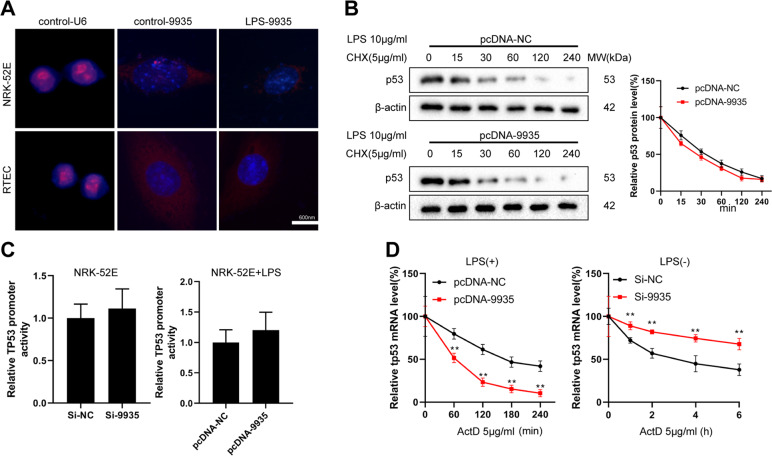
LncRNA 9935 regulated the stability of *Tp53*. **A** Fluorescence in situ hybridization (FISH) was executed to confirm the location of 9935 (red) in NRK-52E cells and rat primary RTECs. Nuclei were stained blue with DAPI. **B** NRK-52E cells were transfected with pcDNA-NC or pcDNA-9935 and then treated with LPS and cycloheximide (CHX; 5 μg/ml). The protein level of p53 was measured at the indicated time points after CHX administration. **C**, **D** NRK-52E cells were divided into four groups: si-NC, si-9935, pcDNA-NC+LPS, and pcDNA-9935+LPS. **C** The promoter activity of *Tp53* was detected by luciferase gene reporter assay. **D** Each group of cells was treated with actinomycin D (AtcD; 5 μg/ml) and the *Tp53* mRNA level was determined by qRT-PCR at the indicated time points. **P < 0.01 vs. pcDNA-NC or si-NC.

Next, we explored the underlying mechanism by which 9935 affected the LPS-engendered apoptosis of RTECs. The FISH assay performed on RTECs and the kidneys of septic AKI rats confirmed that 9935 was mainly localized in the cytoplasm (Fig. 4A and Fig. S2B). As the preceding data showed that 9935 affected both mRNA and the protein levels of p53 (Fig. 2), we assessed the impact of 9935 on p53 from the aspects of protein stability, transcriptional activity, and mRNA stability. As shown in Fig. 4B, the overexpression of 9935 did not affect the degradation of p53 protein in LPS-treated NRK-52E cells. As shown in Fig. 4C, neither 9935 interference nor overexpression changed the *Tp53* promoter activity, which suggested that 9935 was unable to affect the transcription of p53. Interestingly, the results of Fig. 4D showed that the decay of *Tp53* mRNA was facilitated by 9935 overexpression and repressed by 9935 interference. These data suggested that 9935 regulated the mRNA and protein levels of p53 by affecting its mRNA stability.

### 9935 decreased *Tp53* mRNA stability through HuR

**Fig. 5:**
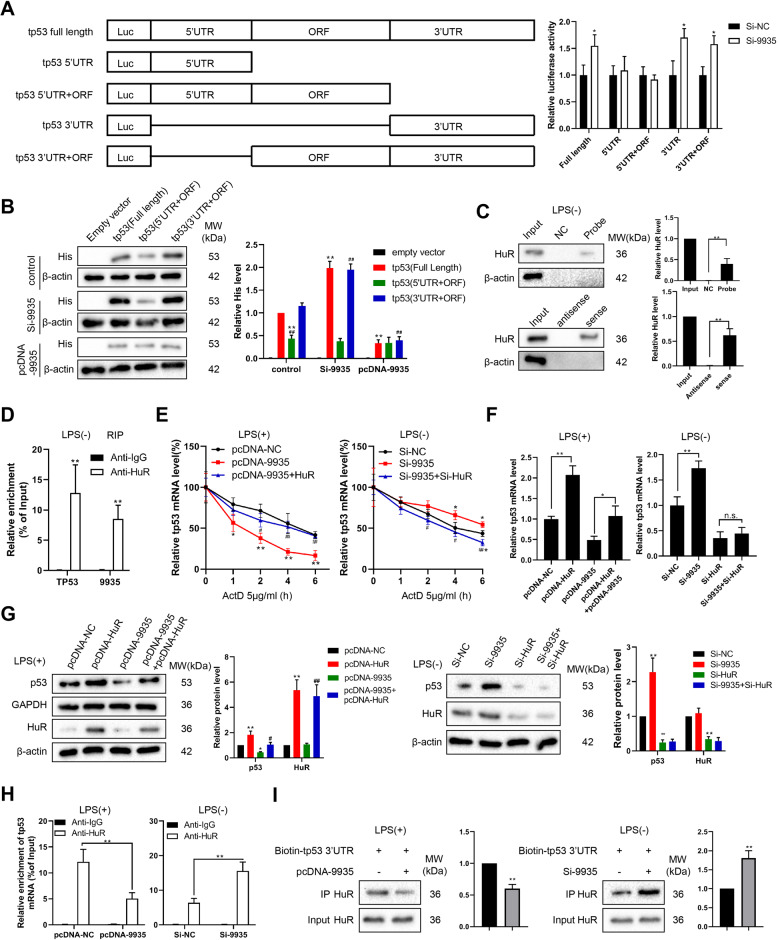
LncRNA 9935 reduced the *Tp53* mRNA stability through human antigen R (HuR). **A** The dual-luciferase reporter assay was performed on si-9935 (or si-NC)-transfected NRK-52E cells using pGL3 reporters that contain various regions of the *Tp53* transcript (full length, 5′UTR, 5’UTR + Open Reading Frame (ORF), 3′UTR, 3′UTR + ORF). *P < 0.05 vs. si-NC. **B** Expression vectors containing p53 ORF in combination with either the 3′UTR, the 5′UTR, or both (named as full length) were generated and labeled with histone (His). Western bolt analysis for histone was performed on NRK-52E cells co-transfected with si-9935/pcDNA-9935 and indicated vectors. Empty vector was served as the negative control. **P < 0.01 vs. control-tp53 (Full length), ##*P* < 0.01 vs. control-tp53 (3′UTR + ORF). **C** Upper: NRK-52E cells were transfected with an antisense biotin-labeled DNA oligomer against 9935 or its negative control (NC, a biotin-labeled sense DNA oligomer probe), followed by the pull-down experiments. The pull-downed complexes were analyzed by western blot with anti-HuR and anti-β-actin antibodies. Below: Lysates of NRK-52E cells were incubated with biotin-labeled 9935 or its antisense RNA, followed by the pull-down experiments. The pull-downed complexes were analyzed by western blot with anti-HuR and anti-β-actin antibodies. **P < 0.01. **D** RNA immunoprecipitation (RIP) assay was performed to examine the combination of HuR and 9935/*Tp53* mRNA in NRK-52E cells. **P < 0.01 vs. Anti-IgG. **E**–**H** NRK-52E cells were transfected with indicated vectors, followed by LPS treatment or normal culture. **E** *Tp53* mRNA stability (*P < 0.05, **P < 0.01 vs. pcDNA-NC/si-NC; #*P* < 0.05, ##*P* < 0.01 vs. pcDNA-9935/si-9935), **F** *Tp53* mRNA level (*P < 0.05, **P < 0.01, n.s. no significant difference), and **G** protein levels of p53 and HuR were detected. *P < 0.05, **P < 0.01 vs. pcDNA-NC or si-NC, #*P* < 0.05, ##*P* < 0.01 vs. pcDNA-9935. **H** RIP assay was performed to detect the influence of 9935 on the combination of HuR and *Tp53* mRNA. **P < 0.01. **I** RNA pull-down assay was performed to detect the influence of 9935 on the combination of *Tp53* 3′UTR and HuR. **P < 0.01 vs. biotin-tp53 3′UTR.

To further investigate whether 5′UTR or 3′UTR of *Tp53* mRNA is indispensable for 9935 to regulate the mRNA stability of *Tp53*, a dual-luciferase reporter assay using the pGL3 reporters, which contained various regions of the p53 transcript (Fig. 5A) was performed. The results depicted that an obvious elevation of luciferase activity was observed in the cells co-transfected with a reporter containing full length/3′UTR/3′UTR + ORF of *Tp53* mRNA and si-9935 (the transfection efficiency of si-9935 was shown in Fig. S2C). Moreover, the expression vectors containing the p53 protein ORF in combination with either 3′UTR or 5′UTR or both (named as full length) were generated and labeled with histone. Compared with control NRK-52E cells, the expression efficiencies of *Tp53* ORF + 3′UTR and full length were higher in 9935-silenced NRK-52E cells and decreased in 9935-overexpressed NRK-52E cells, while the expression efficiency of *Tp53* ORF + 5′UTR was not changed in both 9935-silenced NRK-52E cells and 9935-overexpressed NRK-52E cells (Fig. 5B). Collectively, the above data demonstrated that 9935 might reduce the mRNA stability of *Tp53* through its 3′UTR region. HuR is an RNA-binding protein (RBP) that stabilizes *Tp53* mRNA by binding to the 3′UTR region of *Tp53* [19]. To identify 9935-binding proteins, the RNA pull-down assay was conducted using a biotin-labeled antisense DNA oligomer probe against 9935 and its negative control (biotin-labeled sense DNA oligomer probe). This analysis detected a approximately 36 kDa band (Fig. S2D) and the efficiency and specificity of the 9935 probe were detected by qRT-PCR (Fig. S3A). The subsequent western blot analysis confirmed it was HuR protein (Fig. 5C). Meanwhile, the biotin-labeled 9935, rather than its antisense RNA, was able to pull down endogenous HuR protein in NRK-52E cells (Fig. 5C). The results of the RIP assay shown in Fig. 5D confirmed the combination of HuR and *Tp53*/9935. The overexpression of HuR removed the promoting effect of pcDNA-9935 on *Tp53* decay (Fig. 5E), increased *Tp53* mRNA level (Fig. 5F) and protein expression (Fig. 5G) in LPS + pcDNA-9935-treated NRK-52E cells. HuR silencing eliminated the inhibitory effect of si-9935 on *Tp53* degradation, and the inhibitory effect of si-HuR on the mRNA and protein levels of p53 did not affect by 9935 silencing in normal NRK-52E cells (Fig. 5E–G). As shown in Fig. 5H, the overexpression of 9935 decreased the enrichment of *Tp53* mRNA in the immunoprecipitate of HuR in LPS-treated NRK-52E cells, and the silencing of 9935 increased the enrichment of *Tp53* mRNA in the immunoprecipitate of HuR in normal NRK-52E cells. Meanwhile, the results of the RNA pull-down assay showed that 9935 was unable to directly bind to *Tp53* mRNA (Fig. S3B), which suggested that 9935 indirectly decreased the *Tp53* mRNA stability by reducing the combination of HuR and *Tp53* 3′UTR. Consistent with our speculation, the results of the RNA pull-down showed that 9935 overexpression decreased the amount of HuR pulled down by *Tp53* 3′UTR in LPS-treated NRK-52E cells, and that the 9935 knockdown enriched the amount of HuR pulled down by *Tp53* 3′UTR in normal NRK-52E cells (Fig. 5I).

### 9935 overexpression suppressed the LPS-induced RTEC apoptosis through HuR/p53 axis

**Fig. 6:**
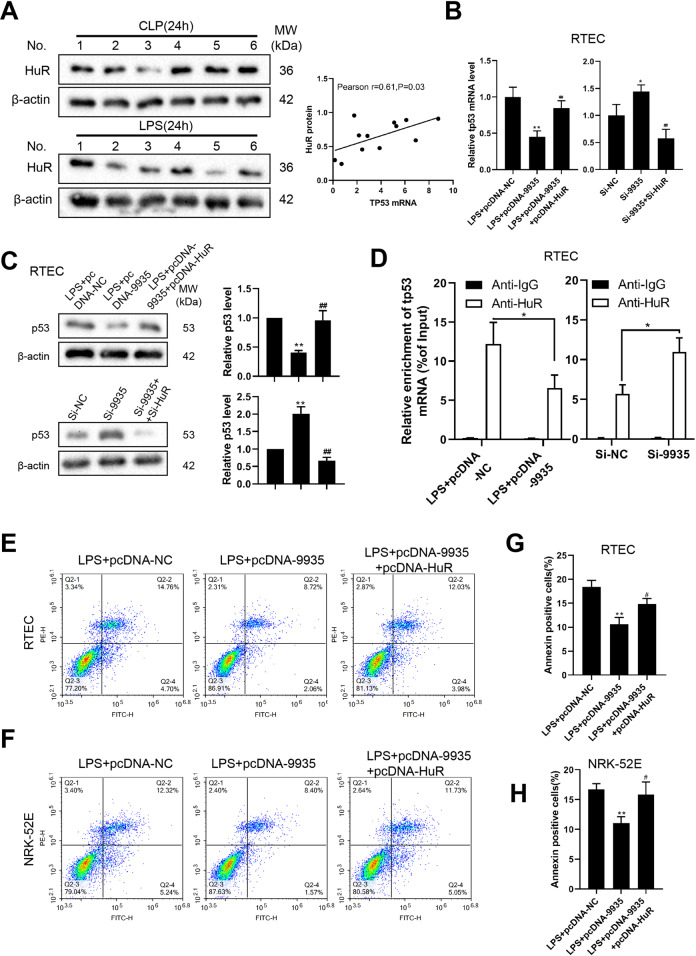
LncRNA 9935 overexpression suppressed LPS-induced RTEC apoptosis through HuR/p53 axis. **A** HuR protein level was measured in the rat model of septic AKI induced by CLP or LPS using western blot. The correlation between the HuR protein level and *Tp53* mRNA was assessed. **B**, **C** The influence of 9935 and HuR on p53 expression was measured in rat primary RTECs. *P < 0.05,**P < 0.01 vs. LPS + pcDNA-NC or si-NC; ##*P* < 0.01 vs. LPS + pcDNA-9935 or si-9935. **D** RIP was performed to detect the influence of 9935 on the combination of HuR and *Tp53* mRNA in RTECs. *P < 0.05. **E**–**H** The influence of 9935 and HuR on LPS-induced cell apoptosis was performed on primary RTECs and NRK-52E cells using flow cytometry. The representative flow scatters plots and quantitative results were shown. **P < 0.01 vs. LPS + pcDNA-NC; #*P* < 0.05 vs. LPS + pcDNA-9935.

We assessed the HuR protein level in the kidney tubules of septic AKI rats induced by CLP or LPS, and found a positive correlation between the HuR protein level and *Tp53* mRNA (Fig. 6A) in the kidney tubules of septic AKI rats. We further confirmed the modulatory effect of 9935 and HuR on p53 expression using rat primary RTECs. As shown in Fig. 6B–D, pcDNA-9935 suppressed the mRNA and protein levels of p53 and decreased the interaction between HuR and *Tp53* mRNA in LPS-treated rat primary RTECs, whereas the suppressive effect of pcDNA-9935 on p53 expression was removed by HuR overexpression. In the rat primary RTECs, the interference of 9935 elevated the mRNA and protein levels of p53 and enhanced the interaction between HuR and *Tp53* mRNA, whereas the promoting effect of si-9935 on p53 expression was abrogated by si-HuR (Fig. 6B–D). In both rat primary RTECs and NRK-52E cells, the overexpression of 9935 decreased the LPS-induced cell apoptosis, whereas the anti-apoptosis effect of pcNDA-9935 was removed by pcDNA-HuR (Fig. 6E–H). The above data indicated that 9935 reduced p53 expression through HuR, thus restraining the p53-mediated RTEC apoptosis.

## Discussion

Although an increasing number of researchers have realized that the apoptosis of RTECs is a crucial contributor to the onset and development of septic AKI [8, 9], the pathological mechanism by which RTEC apoptosis is upregulated during septic AKI is not entirely clear. In the present study, we determined a novel lncRNA, 9935, which was downregulated during the septic AKI progression and its downregulation enhanced the HuR-mediated *Tp53* mRNA stability. The latter led to the increased p53-mediated apoptosis of RTECs. Through overexpressing 9935, the p53-mediated apoptosis of RTECs was efficiently suppressed and the pathological progress of septic AKI was partly slowed down, which suggests the potential therapeutic effect of 9935 on septic AKI.

CLP surgery and LPS injection are the two most commonly practiced methods to model the experimental septic AKI [20]. The CLP procedure leads to peritonitis through exposing the animal to its feces, while LPS administration mimics the hyper inflammation that occurs in early sepsis via activating the immune system [21, 22]. Based on the results of RNA sequencing, we defined 9935 and the other three lncRNAs as DE-Lncs in the rat model of septic AKI induced by CLP. Then, the expression levels of four DE-Lncs were detected in the rat model of LPS-induced septic AKI and LPS-treated NRK-52E cells. The data showed that 9917, 9935, and 1687 expressions were also significantly reduced in the LPS-treated rats and that 9935/9917 overexpression efficiently reduced the c-caspase 3 protein level in LPS-treated NRK-52E cells. As the level change in 9935 was the most significant and the 9935 overexpression suppressed the c-caspase 3 protein level most obviously, we speculated that 9935 may participate in the septic AKI progression by regulating RTEC apoptosis and selected 9935 for further investigation. Consistent with our speculation, further experiments confirmed that 9935 overexpression suppressed LPS-induced RTEC apoptosis in vitro and retarded septic AKI development in both CLP-treated and LPS-treated rats. Thus, we explored the specific mechanism of 9935 in suppressing the apoptosis of RTECs.

The central role of p53 in the apoptosis of RTECs during septic AKI has been confirmed by accumulating evidence. Yang et al. [23] revealed that the elevation of p53 contributed to the G1 cell cycle arrest in a rat model of septic AKI. Li et al. [24] proved that miRNA-186 suppressed the cell apoptosis rate and preserved the physiological structure of renal tubules by targeting the p53 pathway. In our study, the Pearson correlation analysis revealed that 9935 was negatively related to several genes involved in the p53 signaling pathway (*Tp53*, *Fas*, *Bid*, and *Apaf1*). 9935 overexpression reduced the p-p53 protein level and the mRNA levels of the downstream genes of p53. Moreover, in p53-silenced NRK-52E cells, 9935 overexpression was unable to reverse si-p53-suppressed cell apoptosis. These data suggested that 9935 overexpression inhibited the apoptosis of RTECs by suppressing p53 expression. According to previous studies, the central role of p53 in cell apoptosis relies on two pathways: transcription-dependent and transcription-independent [25]. As a transcription factor, nuclear p53 exerts its proapoptotic function by transactivating several of the target genes (e.g., *Puma* and *Apaf1*) [26]. Meanwhile, p53 located in mitochondria can directly interact with the Bcl-2 family, thereby releasing cytochrome C and activating apoptosis [27]. In the present study, the reduced protein level of p-p53 and mRNA levels of the downstream genes of p53 in 9935-overexpressed NRK-52E cells suggested that 9935 overexpression inhibited p53-mediated transcription-dependent apoptosis. In future works, we will further explore the influence of 9935 on p53-mediated transcription-independent apoptosis.

During the experiments, we observed that both the mRNA and protein levels of p53 were suppressed in response to pcDNA-9935 transfection. This phenomenon prompted us to further explore the specific mechanism by which 9935 suppressing the p53 expression level. As reported, several E3 ubiquitin ligases mediated the ubiquitination of p53 and lncRNA is able to regulate the protein levels by affecting its protein stability [28, 29]. However, our data showed that pcDNA-9935 failed to facilitate p53 protein degradation. Neither the 9935 knockdown nor the 9935 overexpression altered the *Tp53* promoter activity. Note that the subsequent experiments revealed that 9935 negatively regulated *Tp53* mRNA stability, and that this modulatory effect of 9935 relied on the 3′UTR region of *Tp53*. As reported, RBPs modulate the half-life of the target transcript through binding to specific RNA sequences, thus changing the profiles of the expressed gene products [30]. Zou et al. [19] reported that HuR, a member of the Hu family of RBPs, directly binds to the 3′UTR region of *Tp53* and stabilizes *Tp53* in intestinal epithelial cells. Several lncRNAs have been reported to affect the expression levels of target proteins by interplaying with RBPs [31–33]. In line with the previous studies, the combination of HuR and *Tp53* was also detected in NRK-52E cells, and the overexpression of 9935 prominently limited the interaction between HuR and the 3′UTR region of *Tp53*. Moreover, the HuR overexpression abrogated the suppressive effect of pcDNA-9935 on the LPS-induced apoptosis of RTECs. This evidence suggested that 9935 overexpression reduced the apoptosis of RTECs by destroying the HuR-enhanced *Tp53* stability. The data of the present study showed that 9935 was directly bound to HuR, and HuR was directly bound to *Tp53* mRNA 3′UTR. In future work, we will further explore the specific binding site of the HuR protein with 9935 and *Tp53* mRNA to confirm whether 9935 suppresses p53 expression by binding competitively to the HuR protein or reducing the binding ability of HuR to *Tp53* mRNA.

**Fig. 7:**
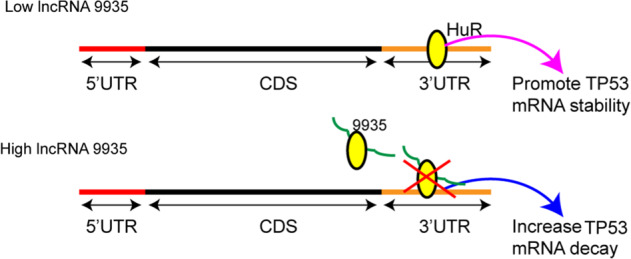
Mechanism figure. 9935 is downregulated during septic AKI progression and its overexpression restrains the combination of HuR and 3′UTR region of *Tp53*, thereby lessening the p53 expression and suppressing the apoptosis of RTECs.

Overall, our research elucidates that 9935 is downregulated during septic AKI progression and its overexpression restrains the combination of HuR and 3′UTR region of *Tp53*, thereby lessening the p53 expression and suppressing the apoptosis of RTECs (Fig. 7). Though a 9935 homologous lncRNA is not found in human cells, 9935 overexpression reduced cell apoptosis and partly suppressed the p53 signaling pathway activation in LPS-treated HK-2 cells (a human RTEC line) (data not shown). This indicates the exogenous supplement of 9953 can alleviate the damage of human RTEC induced by LPS, and highlights the potential clinical significance of 9935.

## Supplementary information

Supplementary Figure 1

Supplementary Figure 2

Supplementary Figure 3
