# Supplementary material for: Upregulation of lncRNA NONRATG019935.2 suppresses the p53-mediated apoptosis of renal tubular epithelial cells in septic acute kidney injury
Source: Cell Death Dis. 2021 Nov 1;12(8):771. doi: 10.1038/s41419-021-03953-9 (PMC8558325; doi:10.1038/s41419-021-03953-9)
Supplement: Supplementary file 1 — Supplementary Figure 1 [file 41419_2021_3953_MOESM1_ESM.docx]

**
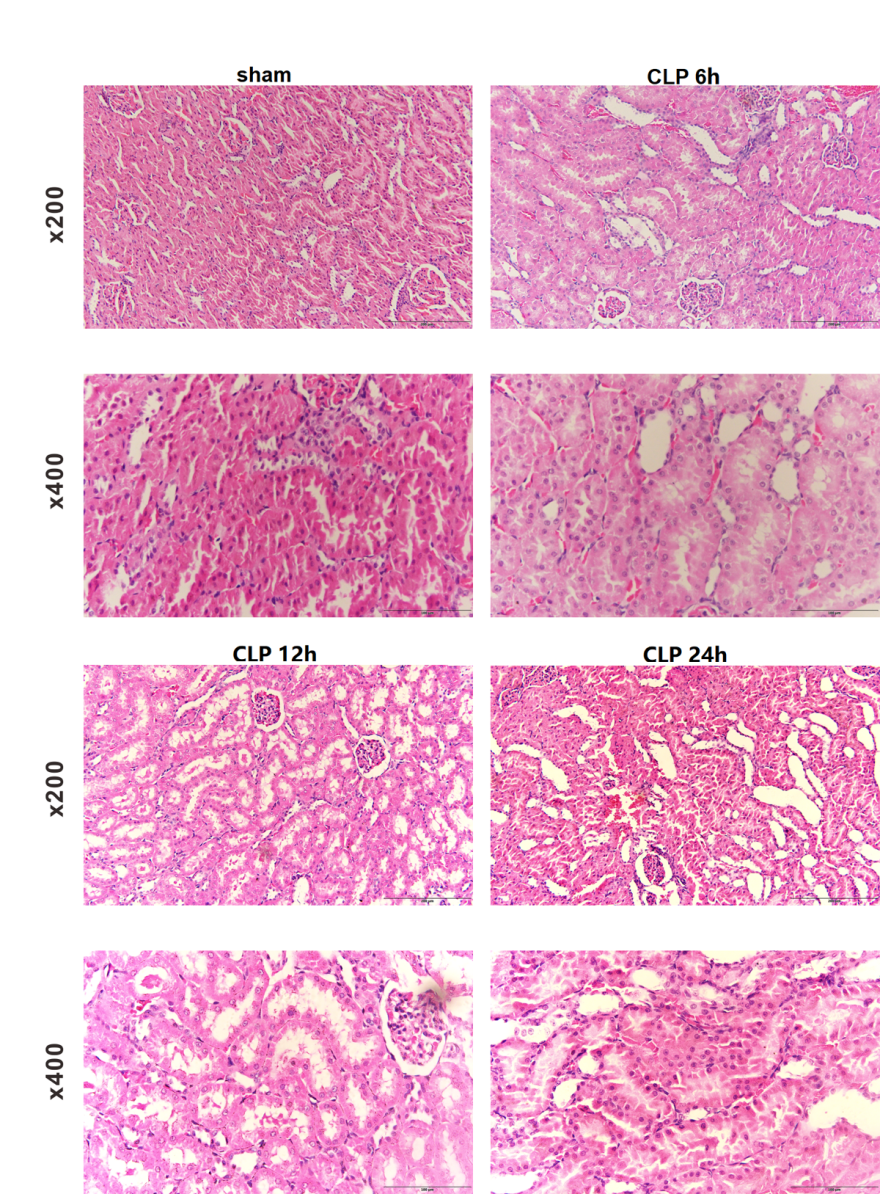
**

**Supplementary Figure 1** Representative images of H&E staining performed on kidneys sections from rats sacrificed at 6/12/24 h after CLP surgery and shame group. sham: *n*=8; CLP 6 h: *n*=6; CLP 12 h: *n*=8; CLP 24 h: *n*=8.
