# Supplementary material for: Upregulation of lncRNA NONRATG019935.2 suppresses the p53-mediated apoptosis of renal tubular epithelial cells in septic acute kidney injury
Source: Cell Death Dis. 2021 Nov 1;12(8):771. doi: 10.1038/s41419-021-03953-9 (PMC8558325; doi:10.1038/s41419-021-03953-9)
Supplement: Supplementary file 2 — Supplementary Figure 2 [file 41419_2021_3953_MOESM2_ESM.docx]

**
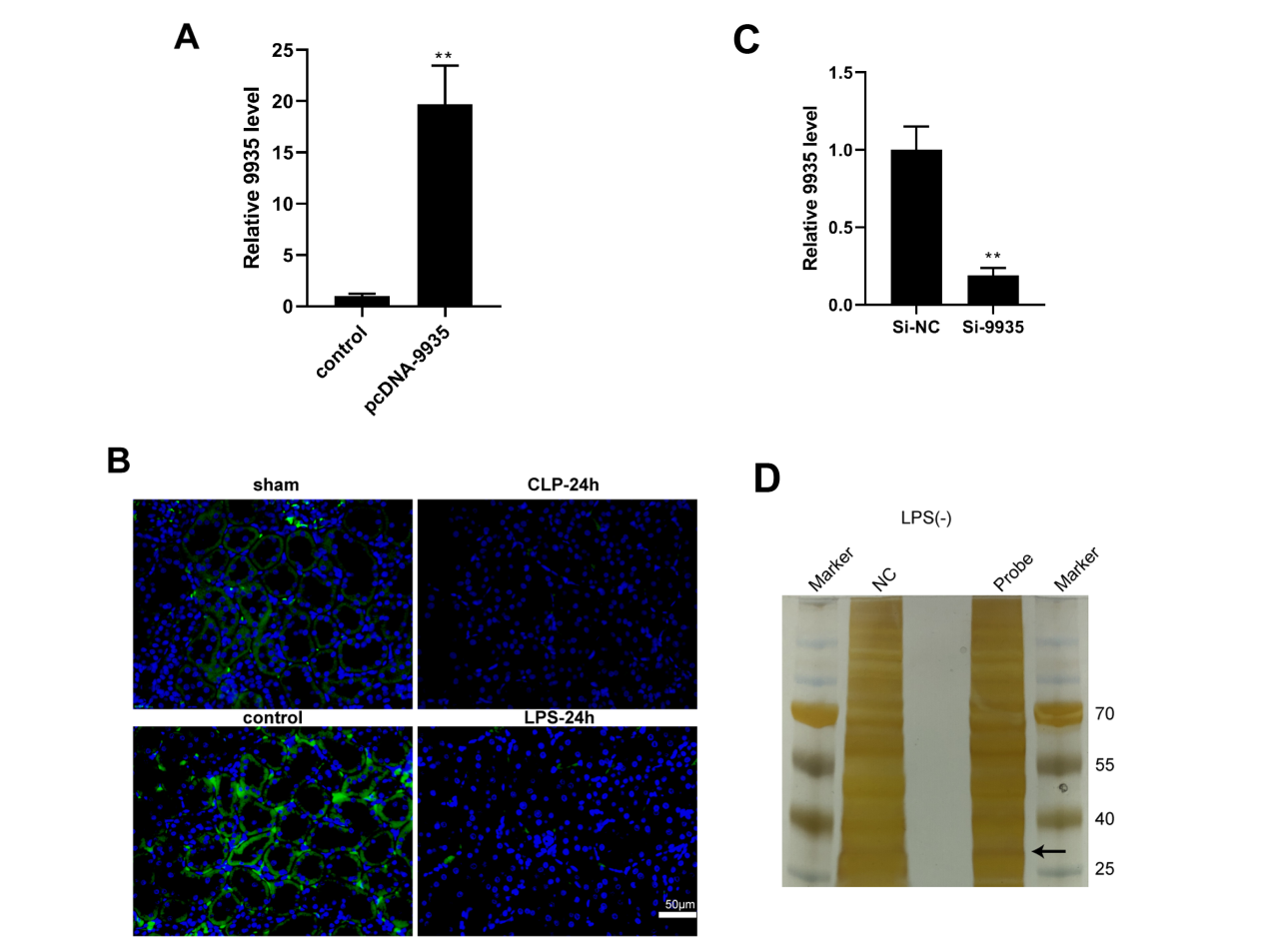
Supplementary Figure 2** (A) The 9935 expression in NRK-52E cells transfected with pcDNA-9935 was measured using qRT-PCR 48 h after transfection. (B) RNA FISH was executed to confirm the location of 9935 (green) in the kidney of the rat model of septic AKI. (C) The 9935 expression in NRK-52E cells transfected with si-NC/si-9935 was measured using qRT-PCR 48 h after transfection. (D) An analysis of RNA-associated proteins in RNA pull-down assay. ***P*<0.01 vs control or si-NC.
