# Supplementary material for: Upregulation of lncRNA NONRATG019935.2 suppresses the p53-mediated apoptosis of renal tubular epithelial cells in septic acute kidney injury
Source: Cell Death Dis. 2021 Nov 1;12(8):771. doi: 10.1038/s41419-021-03953-9 (PMC8558325; doi:10.1038/s41419-021-03953-9)
Supplement: Supplementary file 3 — Supplementary Figure 3 [file 41419_2021_3953_MOESM3_ESM.docx]

**
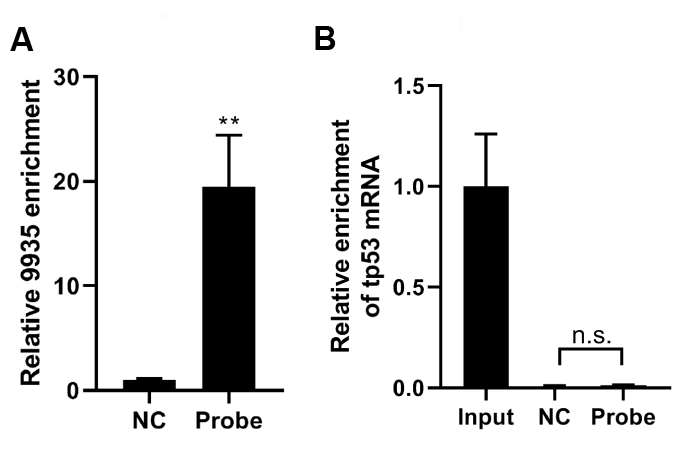
**

**Supplementary Figure 3** (A) The 9935 expression in the complexes pulled down by 9935 biotin-labeled DNA oligomer probe and NC probe were measured using qRT-PCR (***P*<0.01 vs NC probe). (B) The RNA pull-down assay was performed to detect the combination of *Tp53* mRNA and 9935 (n.s.= no significant difference).
